# The impact of *in ovo* injection of cluster bean peptide on hatchability, growth performance, carcass characteristics, digestive enzymes, and blood indices of broiler chickens

**DOI:** 10.1186/s12917-025-04636-9

**Published:** 2025-03-25

**Authors:** Hussein H. El-Fakhrany, Zenat A. Ibrahim, Elwy A. Ashour, Mahmoud Alagawany

**Affiliations:** 1 Poultry Department, Faculty of Agriculture, Zagazig University, Zagazig, 44511 Egypt; 2 Alwadi Farms Poultry Company, El Sheikh Zayed, B1 Capital Business Park, Giza, Egypt

**Keywords:** *In ovo* injection, Cluster bean peptide, Growth performance, Digestive enzymes, Blood indices, Broilers

## Abstract

The administration of bioactive short peptides through *in ovo* feeding can improve the overall health and performance of broiler chickens for the poultry industry. Additionally, bioactive peptides possess biological features that have the potential to be beneficial in preventing many metabolic illnesses; hence, the ingestion of these peptides holds the potential to be advantageous for human health. In light of this, the current work aimed to study the impacts of *in ovo* feeding during the late stages of embryonic development with cluster bean peptide (CBP) on the hatchability, productive performance, lipid profile, liver and kidney functions, immunological response, and antioxidant status of broilers. Six hundred and forty-eight (648) fertilized Ross 308 broiler breeder eggs were used in this study. To remove infertile eggs and dead embryos, the eggs were manually candled on 7 and 17 day of incubation (DOI). On the 18.5th DOI, the eggs were separated into four treatment groups (156 eggs/each), and the first group did not receive any treatment and represented the negative control (NC). Meanwhile, the other treatment groups were injected into the amnion membrane. The second group was only subjected to needle penetration and represented the positive control (PC). The third group was denoted by the letter T1 and received an injection of 1 mg CBP/egg. The fourth group was denoted by the letter T2 and received an injection of 2 mg CBP/egg. *In ovo* feeding by CBP exhibited significant improvements in the body weight of newly hatched chicks, particularly at the 2 mg CBP level. The administration with CBP did not significantly affect the carcass characteristics of 28-day-old broilers. *In ovo*-administrated groups with CBP, higher plasma concentrations of total protein and its fractions were observed at hatch and on day 28 of age. *In ovo* treatment with CBP, blood lipid profile parameters significantly improved at hatch and 28 days of age. Liver and kidney function parameters were improved in response to the *in ovo* administration with CBP in newly hatched chicks and on day 28 of age. Blood levels of glutathione (GSH) and superoxide dismutase (SOD) were considerably higher in the *in ovo*-administered groups with CBP; while levels of malondialdehyde (MDA) were significantly reduced due to CBP administration. The activity of digestive enzymes in blood plasma was decreased in newly hatched chicks but increased in 28-day-old broilers in response to *in ovo* administration with CBP. There was an improvement in the immunological response of hatched chicks from groups injected with CBP, particularly the T2 group (2 mg CBP), as evidenced by increased IgM and IgG levels. The findings presented here indicate that the *in ovo* administration with CBP, specifically at a dosage of 2 mg, improved growth performance and immune and antioxidant functions.

## Introduction

During artificial incubation, *in ovo* injection is an acknowledged technique widely utilized in embryo immunization operations. According to the findings of several studies [1, 17, 61], the administration of exogenous nutritional components to the amniotic sac of bird embryos has the potential to induce the development of both embryos and post-hatched chicks. Most poultry species (e.g., chickens, ducks, geese, ostriches, and quail) are classified as precocial birds, which rapidly increase the metabolic process and oxygen utilization around the hatching period. This surge is essential to fulfill the requirements of endothermy and movement, which are made possible by the transformation from respiration through the chorioallantoic membrane to pulmonary respiration [7, 29]. In the lifecycle of broiler chickens, the most important period is the late period of embryo life and the early period of chick life. In addition, delaying food availability negatively impacts the immune system, increasing their susceptibility to infections and resulting in a lower survival rate [1]. According to Akosile et al. [5], the *in ovo* administration of natural antioxidants is a promising method for enhancing chickens' antioxidant status and overall performance. Recently, there has been a growing trend toward using phytochemical compounds owing to their possible antioxidative activity [6, 28, 39, 56].

Cluster beans (*Cyamopsis tetragonoloba*) are an important annual legume crop in the family *Fabaceace*. It is mainly cultivated for its fodder, vegetables, galactomannan, and green purposes. Cluster beans are guar bean, guari, khutti, and chavlikayi [54]. Its cultivation dates back to Africa, but now it is widely cultivated in India, Brazil, Australia, Pakistan, Malawi, South Africa, Sudan, Zaire, and the USA [10]. India contributes about 80% of the world's cluster bean production. Cluster bean has versatile applications in various industries, including papers, textiles, printing, pharmaceuticals, cosmetics, mining, well drilling, petroleum, natural oil and gas [34]. Green cluster beans are nutritious and eaten as vegetables in some countries. Cluster beans, rich in protein, have great potential as a valuable feed ingredient for monogastric animals [34]. It is also recognized as a medicinal plant with many phytochemicals [25, 47]. Bioactive legume peptides are known to contain short peptides with hydrophilic and hydrophobic amino acid residues, which possess higher inhibitory activity against certain enzymes (Moreno-Valdespino et al., 2020). Among several legume proteins studied, cluster beans were found to have high protein content [42].

The last products obtained from the digestion of proteins in the gastrointestinal system are known as small peptides [48]. They are responsible for numerous varieties of biological functions in animals. The typical composition of these molecules includes two to three amino acids, and their average molecular weight is roughly 300 Daltons. According to Lafarga et al. [35], bioactive peptides are short sequences that contain between two and thirty amino acids (AA). Maestri et al. [41] found that the molecular weight of bioactive peptides ranged from four hundred to two thousand Daltons. However, the bioactivity and bioavailability of bioactive peptides depend on the composition and sequencing of the AA in the peptide [16, 43]. Definitions of these small bioactive peptides vary from study to study. According to Judák et al. [31], several scientists define these peptides as peptides with a molecular weight of less than 1000 Da, while others define them as peptides with a molecular weight of less than 2000 Da. Other publications also classify these peptides as having 15–20 AA. On the other hand, the structure of these peptides is straightforward in contrast to large proteins, which can be composed of several hundred amino acids folded into complicated three-dimensional structures. However, these peptides can exhibit several receptors' activities and play significant roles in several biological processes [31].

A specific concentration of the bioactive peptides is necessary for animals to realize their full potential in terms of performance. According to research conducted by Kogut et al. [33], these peptides have been shown to positively influence the development of the intestinal tract by enhancing its architecture and function, which in turn facilitates the absorption of nutrients. Furthermore, it has been revealed that these bioactive peptides can operate on immunocytes, improving the immune system and disease resistance in animals [45]. As a result, it is essential to supplement chicken diets with bioactive peptides to support the physiological status, encourage growth, and decrease the mortality rates of birds. We hypothesized that cluster bean peptide (CBP), as a bioactive peptide, would play significant roles in the growth of broiler chickens because of its nutritional capacity and biological functions. In light of this hypothesis, we decided to investigate the effects of *in ovo* administration with CBP at the late stage of embryonic development on hatchability and hatched chicks' growth performance, liver and kidney functions, antioxidant status, digestive enzyme activity, and immunological response of broilers.

## Materials and methods

### Treatments

The study was conducted in Alwadi Farms Poultry Company, B1 Capital Business Park, El Sheikh Zayed, Giza, Egypt. Six hundred and forty-eight (648) fertilized Ross 308 broiler breeder eggs were used in this study. The weight of each egg was determined individually, and the average weight of the eggs was 60.83 ± 3 g. All eggs were transferred to the incubator (chick master multi-stage- the capacity was 87,480 eggs) provided by Alwadi Poultry Company, where they were subjected to an incubation protocol consisting of 37.6 ± 0.02 °C and 60.5% RH. To remove infertile eggs (clear eggs) and dead embryos, the eggs were manually candled at 7 and 17 DOI. On the 18.5th DOI, the eggs were distributed into four equal groups of 156 eggs (six replicates of 26 eggs each). The first group received no treatment and represented the negative control (NC) while the other treatment groups were injected into the amnion membrane [2], and the candling was performed on the egg to locate the injection site. The injection site was cleaned and sterilized with 70% ethyl alcohol, and tested material was introduced into the injection site using a graded insulin syringe (1 ml). The second group was only subjected to needle penetration and represented the positive control (PC). The third group was denoted by the letter T1 and received an injection of 1 mg CBP/egg. The fourth group was denoted by the letter T2 and received an injection of 2 mg CBP/egg. The injection site was closed with non-toxic glue, and the eggs were immediately reset in the incubator. After hatching, the chicks were individually weighed and distributed into four groups. Each group consisted of six replicate pens, with 25 birds in each. The percentage of hatchability was calculated by dividing the count of hatched chicks by the count of fertile eggs multiplied by 100.

### Management post-hatched chicks

The floor pens had dimensions of roughly 1.22 m by 2.44 m and were equipped with fresh pine shavings as the litter material. The average temperature was kept at 33 °C for the first week, then dropped to 28 °C during the second week, and finally settled at 25 °C. Throughout the experiment, the farm building was subjected to artificial ventilation, and the relative humidity was kept at a regulated level of 50% ± 5%. The chicks were given full access to commercial basal feeds that were recommended to meet the nutritional needs of broiler chicks during the starter and finisher periods (Table 1). The birds were given full access to fresh water through a nipple drinker system.

**Table 1:** Composition and chemical analysis of the experimental diets (starter, grower and finisher diets)

Items			
	Starter (1–10 d)	Grower (10-20d)	Finisher (1-28d)
Ingredients (%)			
Yellow Corn	52.90	58.30	60.41
Soybean meal (44% CP)	37.3	32.03	31.00
Corn gluten (62% CP)	3.35	3.13	1.45
Soybean oil	1.78	2.38	3.35
Mono Calcium P	1.28	0.93	0.83
Limestone	1.53	1.45	1.38
Vit-min Premix*	0.30	0.30	0.30
Nacl	0.05	0.05	0.05
Sodium sulfate	0.41	0.42	0.41
Ammonium chloride	0.05	0.05	0.05
DL Methionine	0.36	0.31	0.31
L-Lysine	0.36	0.35	0.23
Arginine	0.05	0.05	0.00
Threonine	0.13	0.10	0.08
Choline Chloride 60%	0.15	0.15	0.15
Calculated analysis** (%)			
CP	22.50	20.50	19.00
ME Kcal/kg diet	2900	3000	3100
Ca	0.90	0.80	0.75
P (Available)	0.45	0.36	0.33
Lysine	1.50	1.38	1.31
Methionine	0.62	0.56	0.48
M + C	0.99	0.90	0.80
CF	3.43	3.75	3.68

^*^Growth vitamin and Mineral premix Each 2.5 kg consists of:Vit A 12000, 000 IU; Vit D3, 2000, 000 IU; Vit. E. 10g; Vit k3 2 g; Vit B1, 1000 mg; Vit B2, 49g; Vit B6, 105 g; Vit B12, 10 mg; Pantothenic acid, 10 g; Niacin, 20 g, Folic acid, 1000 mg; Biotin, 50 g; Choline Chloride, 500 mg, Fe, 30 g; Mn, 40 g; Cu, 3 g; Co, 200 mg; Si, 100 mg and Zn, 45 g

^**^ Calculated according to NRC (1994)

### Post-hatch measurements

#### Measurements of growth performance:

A precise scale (0.01 g) was used to measure the weekly feed intake (FI) and body weight (BW). FI was measured every two weeks by weighing and quantifying the residual amounts of feed and then subtracting them from the presented before offering the new ones. The average weight gain (WG) was determined (g/bird) over different intervals for each replicate. The dead birds were recorded daily, and the mortality rate was computed relating to the initial number of live chicks. The average FI was adjusted according to the mortality rate, and the feed conversion ratio (FCR) was estimated by dividing the adjusted FI by WG.

#### Biochemical tests of blood plasma

At hatch and 28 days of age, twelve chicks from each treatment group were chosen at random, then were anesthetized by using intramuscular injection with 1 ml/kg of ketamine xylazine mixture (2:1) and slaughtered by a sharp knife to complete bleeding and the blood was received in heparinized tube. The blood of every two chicks in the same treatment group was received and blended into a single tube, six tubes for each treatment group. Blood plasma was separated from whole blood by centrifuging the tubes at 3500 rpm for 15 min and then stored at −20° C until tested biochemical components were examined. The colorimetric methodology was applied using commercial kits manufactured by Diamond Diagnostics Company (Egypt) for evaluating blood plasma content of protein fractions (g/dL), lipid profile parameters (mg/dL), and kidney function tests (mg/dL), as well as the activity liver enzymes. The plasma protein fractions that were analyzed calorimetrically were total protein and albumin, while globulin concentration was computed for each sample by deducting albumin from total protein. A subsequent calculation was performed to calculate the albumin/globulin ratio (A/G ratio). The blood lipid profile parameters included cholesterol, triglycerides, HDL, and LDL cholesterol. The kidney function tests included plasma levels of urea and creatinine. The liver function tests included the activity (U/L) of Aspartate Transaminase (AST) and Alanine Transaminase (ALT). The blood plasma contents of immunoglobulin M (IgM) and G (IgG) were estimated using ELISA Kits purchased from Abcam company (Catalog No. Ab157692) for IgM and MyBioSource company (Catalog No. MBS260043) for IgG. The calorimetric method was also performed on blood plasma to measure the activity of digestive enzymes (amylase and lipase) and the measurements of antioxidant status (MDA, SOD, and GSH) by using commercial kits that produced by Biodiagnostic Company (Egypt).

#### Carcass characteristic measurements

On day 28 of broilers' age, twelve birds were randomly chosen per each treatment group, then weighed individually and slaughtered. The carcass and the edible internal organs (giblets), *i.e.,* liver, gizzard, and heart, were removed and weighed (absolute weight, g). The total weight of the giblets was computed by summing the gizzard, liver, and heart weights. The total dressing weight was calculated by summing the carcass weights and total giblets. The aforementioned weights were transformed into percentages of the live bird weight.

### Statistics

All data are presented as the means ± _standard error of the mean and were first checked for normality using the D’Agostino-Pearson normality test. Statistical analysis was performed on all gathered data using the GLM procedure of the SAS Software program (SAS, 2004). The model is: Y_ij_ = ‏Ti ‏ + e_ij_, where Y_ij_ = observation, = overall mean, T_i_ = treatment effect, and e_ij_ = random error. The one-way ANOVA test was performed to detect the significance level (P-Value) of using different treatments (4 treatments). If the P ≤ 0.05, the LSD statistic test was employed for all pairwise comparisons among experimental groups.

## Results

**Fig. 1:**
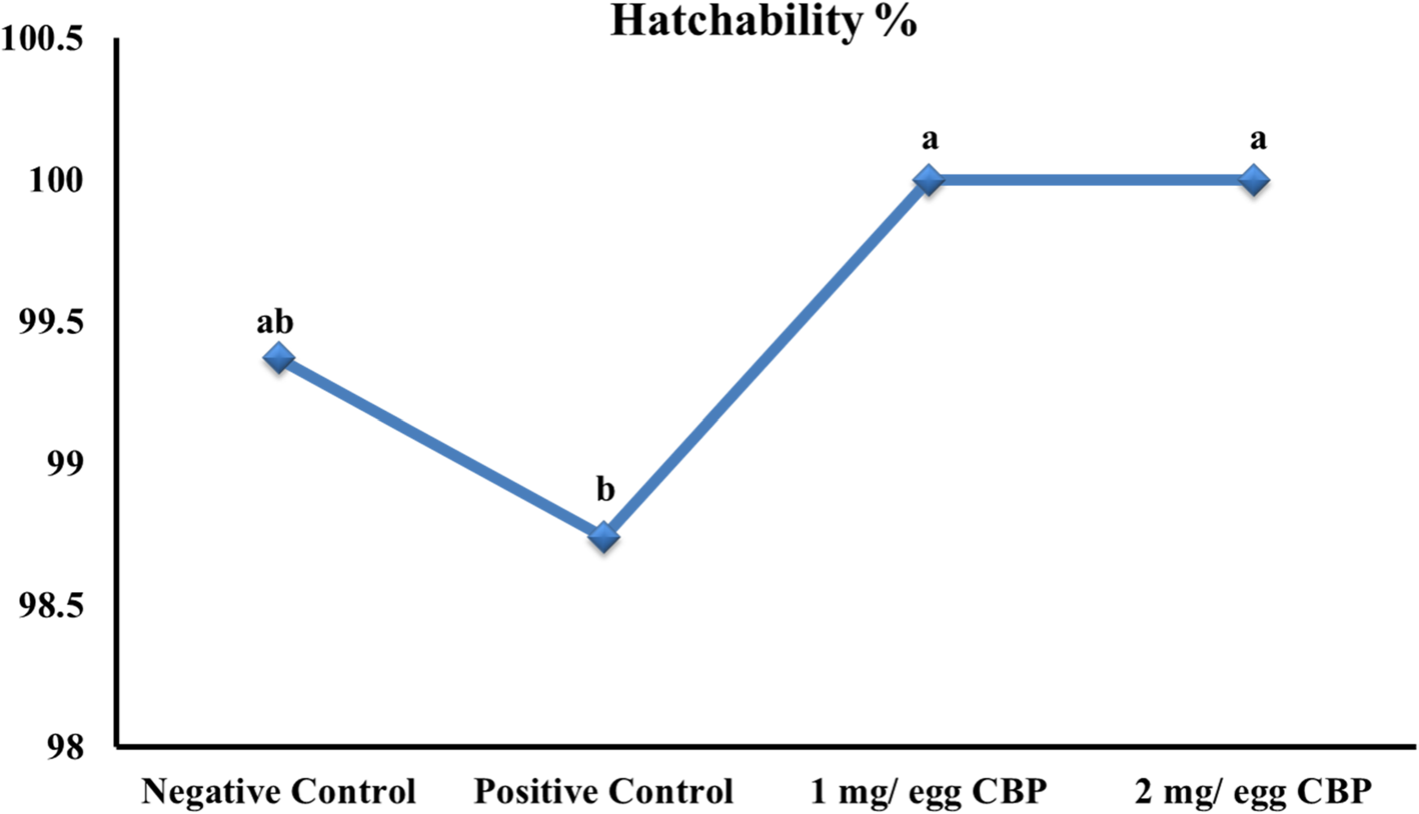
Effect of *in ovo* injection of cluster bean peptide (CBP) on hatchability %

A significant increase in hatchability % and chick weight at hatch was observed in CBP *in ovo*-injected groups compared to the control groups (Table 2, Fig. 1). The BW of broiler chicks at hatch was significantly improved in response to in Ovo injection with 1 mg of CBP compared to all other groups. The administration of 1 mg CBP was more effective than 2 mg in 0 days. But, at 14 and 28 days of age, the *in ovo* administration with different levels of CBP did not significantly affect broiler chickens' BW. The WG followed a similar pattern to BW, indicating no significant differences among trial groups across all trial periods. The *in ovo* administration with different levels of CBP did not exhibit any significant changes in the FI and FCR of broiler chickens.

**Table 2:** Impact of *in ovo* injection of Cluster bean peptide (CBP) on growth performance of broilers

Item	Treatments				*P* value
	Negative Control	Positive Control	1 mg/ egg CBP	2 mg/ egg CBP	
Live body weight (g)					
0	39.51 ± 0.01^b^	38.91 ± 0.14^c^	40.70 ± 019^a^	39.50 ± 0.09^b^	0.000
14 d	400.35 ± 9.20	416.73 ± 1.63	402.44 ± 10.93	410.71 ± 9.57	0.542
28 d	1196.66 ± 1.66	1196.85 ± 3.64	1199.92 ± 5.71	1204.77 ± 8.72	0.718
Body weight gain (g/bird)					
0–14 d	360.83 ± 9.19	377.82 ± 1.49	361.74 ± 10.76	371.21 ± 16.67	0.486
14–28 d	367.90 ± 7.54	796.32 ± 3.17	780.12 ± 16.34	797.48 ± 12.58	0.677
0–28 d	1157.15 ± 1.65	1157.94 ± 3.55	1159.22 ± 5.83	1165.26 ± 8.81	0.739
Daily feed intake (g/bird/day)					
0–14 d	35.97 ± 1.89	36.82 ± 0.99	38.10 ± 3.90	37.41 ± 3.94	0.962
14–28 d	110.92 ± 0.87	113.15 ± 1.97	115.06 ± 1.97	110.65 ± 0.25	0.193
0–28 d	73.45 ± 0.667	74.98 ± 1.44	76.58 ± 2.74	74.03 ± 1.85	0.659
Feed conversion ratio (g feed/g gain)					
0–14 d	1.36 ± 0.06	1.47 ± 0.03	1.42 ± 0.13	1.41 ± 0.18	0.925
14–28 d	1.95 ± 0.00	2.03 ± 0.04	2.02 ± 0.05	1.95 ± 0.03	0.324
0–28 d	1.77 ± 0.01	1.81 ± 0.03	1.85 ± 0.07	1.78 ± 0.04	0.676

^a^^−^^b^Means within row followed by different superscripts are significantly different (*P* < 0.05)

^1^Overall treatment *P*-value

As demonstrated in Table 3, the carcass traits (carcass, dressing, heart, liver, gizzard, and giblets) as a ratio of the pre-slaughter weight, on day 28 of broiler age, was not changed significantly in response to in Ovo feeding with CBP.

**Table 3:** Impact of *in ovo* injection of Cluster bean peptide (CBP) on carcass traits of broilers

Item (%)	Treatments				*P* value
	Negative Control	PC	1 mg/ egg CBP	2 mg/ egg CBP	
Carcass	75.17 ± 1.18	74.87 ± 0.25	74.70 ± 0.92	76.14 ± 0.35	0.593
Dressing	79.47 ± 1.20	78.71 ± 0.21	78.73 ± 0.00	78.73 ± 0.00	0.772
Liver	2.96 ± 0.05	2.51 ± 0.06	2.42 ± 0.12	2.51 ± 0.20	0.059
Heart	0.55 ± 0.01	0.54 ± 0.02	0.58 ± 0.05	0.50 ± 0.03	0.420
Gizzard	0.78 ± 0.04	0.78 ± 0.04	1.03 ± 0.09	0.88 ± 0.07	0.087
Giblet	4.29 ± 0.01	3.84 ± 0.12	4.05 ± 0.019	3.89 ± 0. 26	0.232

Means within row followed by different superscripts are significantly different (*P* < 0.05)

^1^Overall treatment *P*-value

The effect of egg injection with CBP on blood plasma levels of different protein fractions at the hatch is provided in Table 4. Total proteins, albumin, and globulin levels were superior in response to in Ovo injection with 1 mg of CBP compared to all other groups at 0 days. The in Ovo administration with different levels of CBP did not exhibit any significant effect on the A/G ratio of newly hatched chicks. AT 28 days of broiler age, plasma total proteins, albumin, and A/G ratio were considerably (P < 0.05) increased due to the *in ovo* injection with 1 mg CBP, compared to all other groups (Table 5). In addition, plasma globulin concentrations were considerably greater in the positive control group compared to the other groups (Table 5).

**Table 4:** Impact of *in ovo* injection of Cluster bean peptide (CBP) on blood biochemistry of chicks after hatch (at 0 days)

Item^1^	Treatments				*P*-value
	Negative Control	Positive Control	1 mg/ egg CBP	2 mg/ egg CBP	
Liver functions					
TP (g-dl)	3.05 ± 0.100^bc^	2.71 ± 0.07^c^	4.03 ± 0.07^a^	3.32 ± 0.14^b^	0.000
ALB g-dl	1.63 ± 0.04^ab^	1.53 ± 0.08^b^	2.09 ± 0.14^a^	1.95 ± 0.12^ab^	0.020
G (g-dl)	1.42 ± 0.09^ab^	1.18 ± 0.04^b^	1.94 ± 0.14^a^	1.36 ± 0.22^ab^	0.027
A/G ratio	1.16 ± 0.07	1.30 ± 0.11	1.10 ± 0.15	1.55 ± 0.39	0.515
AST (U-L)	17.13 ± 1.26^b^	25.40 ± 1.37^a^	10.51 ± 1.44^c^	10.38 ± 1.35^c^	0.000
ALT (U-L)	151.63 ± 1.99^ab^	164.05 ± 4.14^a^	132.67 ± 3.65^c^	141.54 ± 5.47^bc^	0.003
Kidney functions (mg-dl)					
Creatinine	0.83 ± 0.02^ab^	0.93 ± 0.02^a^	0.76 ± 0.02^bc^	0.69 ± 0.03^c^	0.002
Urea	3.07 ± 0.09^c^	5.63 ± 0.32^a^	4.15 ± 0.15^b^	3.42 ± 0.05b^c^	0.000
Lipid profile (mg-dl)					
TC	243.82 ± 6.40^b^	272.97 ± 3.69^a^	204.55 ± 9.40^c^	169.59 ± 5.49^d^	0.000
TG	144.10 ± 3.52^ab^	153.35 ± 3.86^a^	132.95 ± 4.51^b^	107.87 ± 4.93^c^	0.000
HDL	45.43 ± 1.73	48.99 ± 1.17	46.63 ± 0.62	43.71 ± 0.95	0.071
LDL	169.57 ± 8.35^a^	193.31 ± 5.48^a^	131.32 ± 9.12^b^	104.30 ± 5.22^b^	0.000
VLDL	28.81 ± 0.70^ab^	30.66 ± 0.77^a^	26.58 ± 0.90^b^	21.57 ± 0.98^c^	0.000
Immunity (ng-ml)					
IgM	522.55 ± 19.32^b^	680.00 ± 19.35^a^	310.67 ± 14.15^d^	402.31 ± 12.50^c^	0.000
IgG	587.07 ± 14.45^a^	653.64 ± 35.63^a^	408.60 ± 15.75^b^	485.71 ± 13.78^b^	0.000
Oxidative stress and antioxidants					
MDA nmol-ml	6.815 ± 0.24^b^	10.145 ± 0.51^a^	3.79 ± 0.21^c^	3.14 ± 0.18^c^	0.000
GSH ng-ml	71.31 ± 2.62^b^	37.38 ± 6.15^c^	114.16 ± 5.98^a^	88.77 ± 5.29^b^	0.000
SOD U-ml	92.26 ± 4.83^a^	55.34 ± 3.23^b^	114.93 ± 9.10^a^	87.76 ± 8.37^a^	0.002

^a^^−^^d^Means within row followed by different superscripts are significantly different (P < 0.05)

^1^*TP* total protein; *ALB* albumin; *G* globulin; *ALT* alanine aminotransferase; *AST* aspartate aminotransferase, *TC* total cholesterol; *TG* triglycerides; *HDL* high density lipoprotein; *LDL* low density lipoprotein; *VLDL* very low density lipoprotein, ^1^*SOD* superoxide dismutase; *MDA* malondialdehyde; *GSH* Glutathione reduced; *IgG* immunoglobulin G: *IgM* immunoglobulin M

**Table 5:** Impact of *in ovo* injection of Cluster bean peptide (CBP) on blood biochemistry of chickens at 28 days of age

Items	Treatments				*P* value
	Negative Control	Positive Control	1 mg/ egg CBP	2 mg/ egg CBP	
Liver functions					
TP (g-dl)	3.36 ± 0.12^b^	3.84 ± 0.04^a^	3.97 ± 0.08^a^	2.10 ± 0.11^c^	0.000
ALB g-dl	1.71 ± 0.08^b^	1.64 ± 0.03^b^	2.49 ± 0.13^a^	1.14 ± 0.07^c^	0.000
G (g-dl)	1.65 ± 0.15^b^	2.19 ± 0.00^a^	1.48 ± 0.21^bc^	0.96 ± 0.04^c^	0.002
A/G ratio	1.06 ± 0.15^ab^	0.75 ± 0.01^b^	1.78 ± 0.36^a^	1.19 ± 0.02^ab^	0.035
AST (U-L)	15.88 ± 1.36^b^	13.05 ± 0.74^b^	16.76 ± 4.40^b^	28.94 ± 2.02^a^	0.010
ALT (U-L)	122.73 ± 6.02^b^	116.47 ± 3.79^b^	130.12 ± 5.79^b^	173.70 ± 3.92^a^	0.000
Kidney functions (mg-dl)					
Creatinine	0.67 ± 0.02^b^	0.59 ± 0.02^b^	0.39 ± 0.02^c^	0.95 ± 0.01^a^	0.000
Urea	2.88 ± 0.22^b^	2.22 ± 0.22^bc^	1.52 ± 0.14^c^	5.85 ± 0.20^a^	0.000
Lipid profile (mg-dl)					
TC	170.69 ± 6.22^a^	144.19 ± 4.65^b^	125.38 ± 3.99^b^	125.05 ± 3.13^b^	0.000
TG	142.61 ± 4.54^a^	121.88 ± 5.66^b^	92.46 ± 2.78^c^	153.57 ± 3.36^a^	0.000
HDL	46.64 ± 0.62	47.45 ± 2.51	48.53 ± 1.61	43.58 ± 2.07	0.333
LDL	95.52 ± 7.48^a^	72.42 ± 1.41^b^	58.36 ± 5.39^b^	59.02 ± 4.98^b^	0.003
VLDL	28.52 ± 0.90^a^	24.37 ± 1.13^b^	18.48 ± 0.55^c^	18.91 ± 0.65^c^	0.000
Immunity (ng-ml)					
IgM	315.14 ± 18.46^c^	232.51 ± 11.52^c^	497.48 ± 3.66^b^	606.58 ± 46.55^a^	0.000
IgG	481.27 ± 19.72^b^	410.97 ± 44.59^b^	626.66 ± 14.52^a^	684.97 ± 14.07^a^	0.000
Oxidative stress and antioxidants					
MDA nmol-ml	2.06 ± 0.10^a^	1.43 ± 0.24^b^	0.63 ± 0.08^c^	0.86 ± 0.02^c^	0.030
GSH ng-ml	125.56 ± 5.48^b^	157.86 ± 6.17^ab^	212.37 ± 12.38^a^	179.36 ± 28.95^ab^	0.001
SOD U-ml	124.81 ± 4.50^c^	154.08 ± 3.86^b^c	197.00 ± 7.29^b^	260.76 ± 26.22^a^	0.000

^a^^−^^c^Means within row followed by different superscripts are significantly different (P < 0.05)

^1^*TP* total protein; *ALB* albumin; *G* globulin; *ALT* alanine aminotransferase; *AST* aspartate aminotransferase, *TC* total cholesterol; *TG* triglycerides; *HDL* high density lipoprotein; *LDL* low density lipoprotein; *VLDL* very low density lipoprotein, ^1^*SOD* superoxide dismutase; *MDA* malondialdehyde; *GSH* Glutathione reduced; *IgG* immunoglobulin G: *IgM* immunoglobulin M

Data in Tables 4 and 5 illustrate the measurements of liver and kidney function of broiler chickens in response to the *in ovo* injection with CBP. Based on these findings, *in ovo* administration with CBP exhibited a considerable drop in the activities of plasma AST and ALT and the plasma creatinine and urea levels of newly hatched chicks, and the PC group showed the highest value of them (Table 4). While at 28 days of age, plasma AST, ALT, creatinine, and urea were considerably (*P* < 0.05) increased due to the *in ovo* injection with 2 mg CBP, compared to all other groups (Table 5).

Lipid profile parameters of newly hatched chicks as influenced by in Ovo injection with CBP are tabulated in Table 4. A substantial decrease (*P* < 0.05) in blood plasma cholesterol and triglyceride levels was achieved due to the *in ovo* administration with CBP. The lowest values were recorded in the 2 mg CBP *in ovo*-administered group. Furthermore, notable variations (*P* < 0.001) in LDL levels were detected among the different treatments. The 2 mg CBP *in ovo*-administered group exhibited the lowest concentration of LDL in comparison to the other experimental groups. Despite the non-statistical differences among experimental groups in blood plasma HDL levels, CBP-treated groups (T1 and T2) had the highest concentrations of HDL when compared to the control groups. At 28 days of age, a substantial decrease in plasma levels of cholesterol and triglycerides was detected due to the *in ovo* administration of CBP (Table 5). The T1 group, which received 1.0 mg of CBP per egg, exhibited the lowest levels of cholesterol and triglycerides. In addition, notable (*P* < 0.001) variations in LDL levels were recorded among the different treatments. The T1 group exhibited the lowest concentration of plasma LDL when compared to the other treatment groups. Despite the non-significant effect of treatments on plasma HDL levels, T1 and PC had the highest concentrations of HDL when compared to the different treatments at 28 days of age.

The measurements of the antioxidant status of newly hatched chicks in response to the *in ovo* injection with CBP are shown in Table 4. The blood values of GSH were found to be higher (*P* < 0.05) in the *in ovo* injected with 1 mg CBP, in comparison with the other groups. The activity of SOD was found to be higher (*P* < 0.05) in the negative and the CBP *in ovo*-administrated groups, in comparison with the positive control. On the other hand, it was observed that the levels of blood plasma MDA were considerably lower (*P* < 0.01) in the CBP *in ovo*-administrated groups than in control groups. The T2 group was found to have the lowest value of plasma MDA among the experimental groups. Similarly, on the 28th day of age, the blood plasma levels of GSH and activity of SOD were found to be statistically higher in the CBP-treated groups than in the control groups (Table 5). Conversely, plasma MDA was found at the lowest (*P* < 0.01) level in the T1 group, followed by the T2 group, compared to the control groups.

The effect of *in ovo* injection with CBP (1 and 2 mg/egg) on plasma levels of immunoglobulins (IgM and IgG) of newly hatched chicks is illustrated in Table 4. A noteworthy reduction (*P* < 0.01) in the plasma levels of IgM and IgG was estimated in CBP-treated groups compared to both control groups. Contrarily, on day 28 of age, plasma IgM and IgG were at the highest levels (*P* < 0.01) in the T2 group, followed by the T1 group (Table 5).

**Fig. 2:**
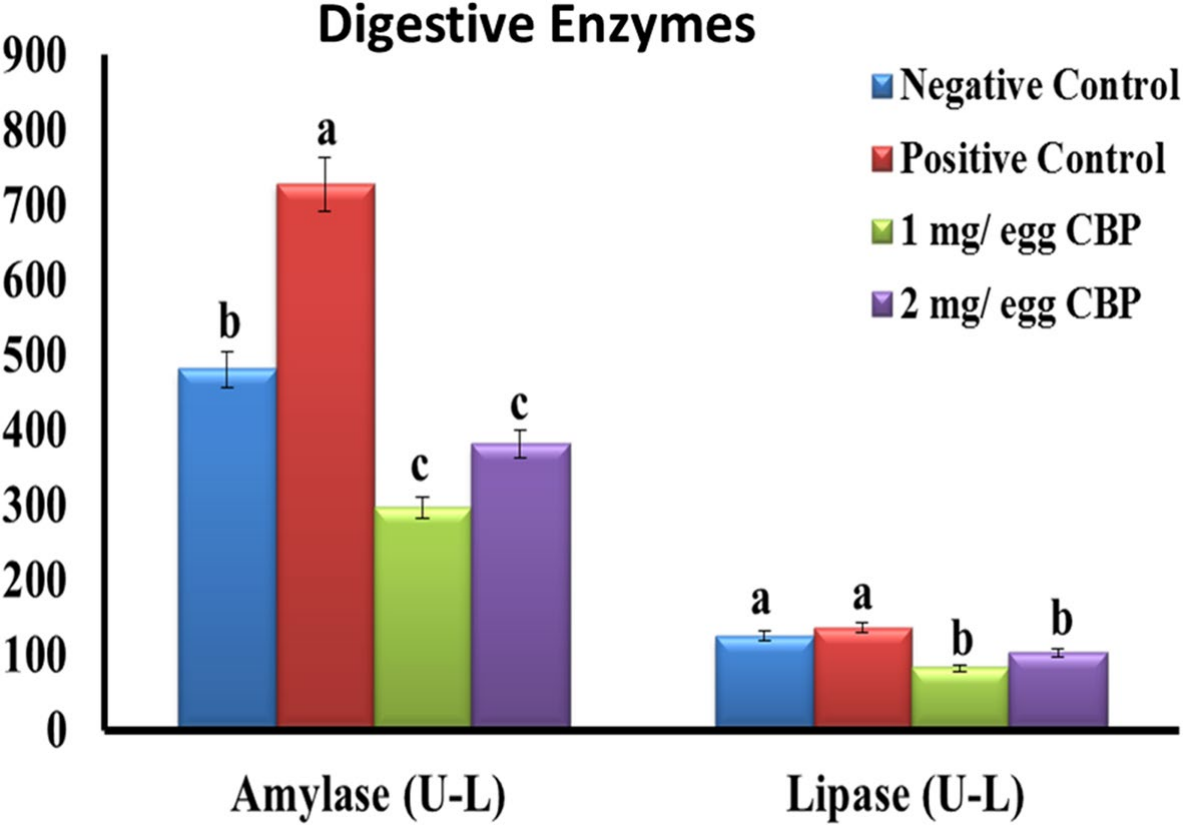
Effect of *in ovo* injection of cluster bean peptide (CBP) on digestive enzymes at hatch (0 day)

**Fig. 3:**
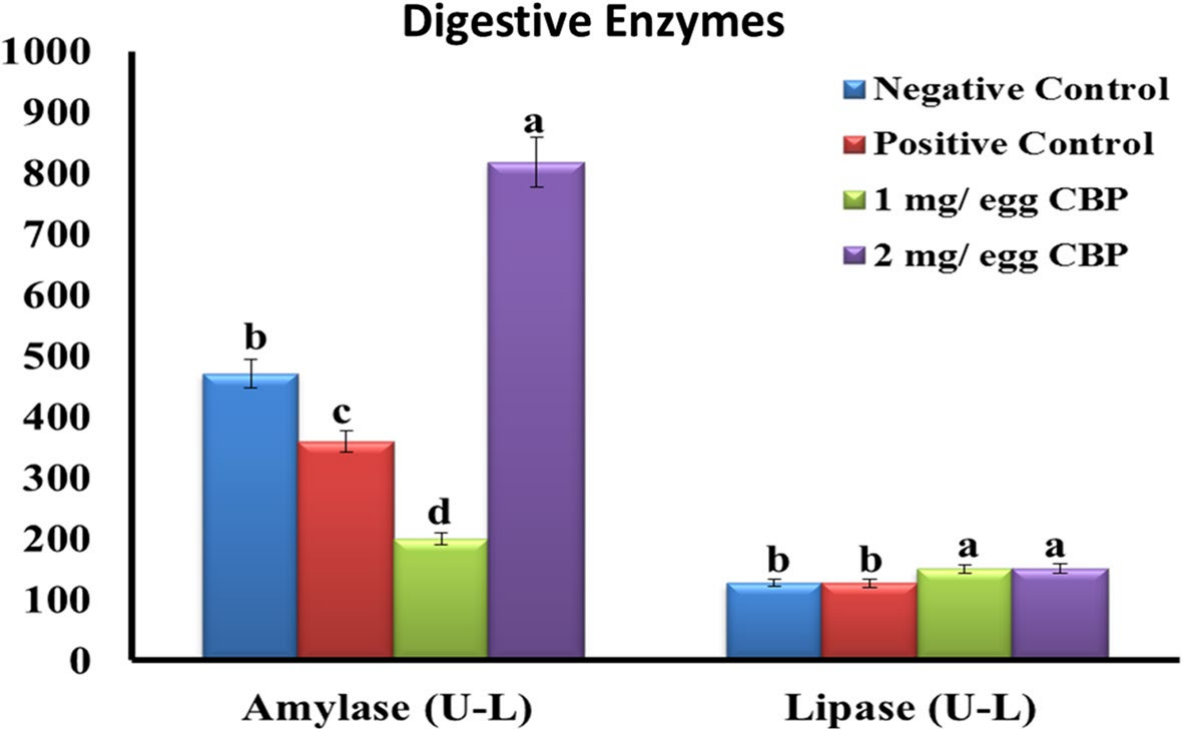
Effect of *in ovo* injection of cluster bean peptide (CBP) on digestive enzymes at 28 days of age

The influence of egg injection with 1 and 2 mg CBP on the activity of digestive enzymes of newly hatched chicks is presented in Fig. 2. Based on these findings at hatch, the activities of amylase and lipase enzymes were lower (*P* < 0.05) in CBP-treated groups than in control groups. At the market age, the levels of amylase in plasma were found to be statistically greater in the T2 group (2 mg of CBP/egg) in comparison to the other groups (Fig. 3). The lipase enzyme activity was higher (*P* < 0.05) in CBP-treated groups than in the control groups.

## Discussion

Compared to other groups, the hatchability of eggs injected with CBP was significantly improved. According to the findings of Perumalla and Hettiarachchy [46], the polyphenolic chemicals that are found in CBP have the potential to assist embryos in overcoming oxidative damage during the hatching process and to inhibit pathogenic bacteria. Furthermore, according to Surai [52], incorporating nutrients with antioxidant properties can boost the antioxidant state and safeguard the tissues and cells from the harmful effects of oxidative damage. However, injecting an egg could be a mild stressor during embryogenesis [19, 23]. The injection procedure may provide hazards to the embryo environment, which might lead to a deleterious effect on the survival rate of the embryo and reduce its ability to hatch [15]. In a study by Ebrahimi et al. [20], injecting fertile eggs with antioxidants through the early period of embryogenesis decreased hatched chicks' number and percentage. These findings were affirmed in the present study. Previous studies have demonstrated conflicting results regarding the effect of in-Ovo injection on hatchability. De Oliveira et al. [18] and Ohta and Kidd [44] attributed these variations to several factors, such as the injected solution concentration, nature and type, the injection time and site (air sac or embryonic membranes), and the means of administration. All of these factors play a significant role in deciding the effectiveness of *in ovo* feeding. The current findings agreed with those of Amina et al. [8], who demonstrated that injecting the fertile egg yolk sac with resveratrol (50 μg/egg) on the 14th day increased the percentage of hatched chicks. The reduction in the formation of damaging free radicals, which can seriously harm the cell membrane integrity, may be responsible for the increased hatchability percentage in resveratrol-injected eggs. Schaal [50] has postulated that *in ovo* injection may increase the consumption of lipids, which could enhance the hatchability percentage. In addition, Khaligh et al. [32] demonstrated that injecting flavonoids in the embryonic amnion membrane on day 18 of incubation increased the hatchability percentage compared to non-injected eggs.

According to Sklan et al. [51], the weight of newly hatched chicks significantly impacts the final weight of broilers. In addition, Wilson [57] declared that broiler marketing weight increased by 8–13 g for every gram increase in the weight of newly hatched chicks. Uni et al. [55] found that egg injection with different types of carbohydrates in the amnion membrane at 17.5 DOI yielded higher chick weight at hatch by about 5–6% than the control. Furthermore, they observed that the increase of newly hatched chick weight by about 2 g in response to in-Ovo feeding increased chick weight at 25 days of age by about 50–60 g. The present experiment coincides with the findings of Amina et al. [8], who found that the weight of chicks at the time of hatch was statistically improved in response to injecting egg with 50 μg resveratrol at 14 DOI, in contrast to NC and PC groups. According to the findings of Choi et al. [14], reducing the oxidative stress associated with the hatching process could potentially lead to increased hatch weights and enhanced post-hatch performance. This would be accomplished by shielding skeletal muscle stem cells from the damaging effects of oxidative stress. In addition, Hajati et al. [26] discovered that the performance of hatched chicks was improved in response to *in ovo* injection with a grape seed extract, which acts as an antistressor throughout the incubation period. According to Liang and Kitts [37] and Lu et al. [38], the active components of CBP, like gallic acid and chlorogenic acid, have antioxidant, antimicrobial, and anti-inflammatory activities [58].

Furthermore, it has been demonstrated that gallic acid or its derivatives heighten the villus height-to-crypt depth ratio in broilers, which indicates improved intestinal architecture and nutrient digestion and absorption [49]. For additional information, gallic acid exhibits antimicrobial activity against harmful bacteria by modifying the features of their cell membrane. According to Yang et al.'s research from 2021, this substance not only reduces oxidative stress and inflammatory reactions but also promotes favorable changes in the microbiota and metabolites of the intestinal tract, which may be helpful to the health of both the host and the intestinal tract. Chlorogenic acid (CGA) has been demonstrated to regulate the microbiota in the chicken's gut, improve the function of the barrier, and alter immunological activity [59]. The improvement in the performance of chlorogenic acid-administered broilers could be attributed to the improvement in immunological functions [62], digestive enzyme activity and nutrients' digestion and absorption [12], intestinal integrity and function [13], digestive tract microbiota [11], and antioxidant status. According to the results of our investigation, the enhancement of growth performance continues till the market age. The improvement in body weight, weight gain, and feed utilization could be due to the improvement in antioxidant and immunological functions at the time of hatch, which remained stable until the animals reached market age.

According to Erinle et al. [22], blood analysis is an important indicator to accurately evaluate the animals' health and physiology. There is a rareness of published publications regarding the effect of *in ovo* administration with CBP on the plasma biochemical indices of broiler chickens. Total protein and its fractions in blood plasma are thought to be indicators of nutritional status and protein metabolism, and immunoglobulins are crucial constituents of these fractions [9]. Our results in the current work revealed that blood plasma levels of total protein and its fractions were dramatically raised in response to *in ovo* injection with 1 mg of CBP, indicating that the injection had a beneficial effect on protein metabolism. In addition, this effect suggests that the treatment with CBP may have a favorable effect on the activity of the liver and indicates that the immune system was strengthened. Noteworthy, the results of liver function tests and immunoglobulin levels in the present study support these suggestions. In the current study, liver and kidney function measurements significantly improved in response to *in ovo* administration with CBP. This effect could be related to the antioxidant, antimicrobial, and anti-inflammatory properties of the active components of CBP, like gallic acid and chlorogenic [37, 38]. These properties could yield protective effects on the liver and kidney tissues. Judák et al. [31] stated that bioactive peptides like CBP could exhibit several receptors' activities and play significant roles in several biological processes, including liver and kidney functions.

Our findings demonstrated that *in ovo* injection with CBP has a beneficial impact on lipid metabolism. The 2 mg CBP-administrated group showed the smallest values of triglycerides and cholesterol and showed the greatest values of HDL in comparison to the other experimental groups. Aditya et al. [4] attributed these beneficial impacts to the presence of anthocyanin in CBP. Also, such action can be related to the polyphenol compounds (epicatechin, gallic acid, and catechin), which function by blocking the action of pancreatic cholesterol esterase that is responsible for breaking down cholesterol esters in the diet, resulting in the release of free cholesterol after digestion. According to Hajati et al. [27], these phenols also bind to bile acid, decreasing the cholesterol solubility in micelles and reducing the amount of absorbed cholesterol by the body via the digestive system. According to Li et al. [36], flavonoids most certainly significantly lowered cholesterol levels. It was also reported by Amina et al. [8] that egg injection with resveratrol substantially decreased (P < 0.001) blood cholesterol of the hatched chicks. In addition, Feng et al. [24] found that supplementing layers' feeds with different levels of resveratrol (0.5, 1.0, 2.0, and 4.0 g/kg feed) significantly reduced circulatory cholesterol levels. In addition, Abu Hafsa and Ibrahim [3] discovered that adding 10, 20, and 40 g grape seeds/kg of broiler diet exerted lower blood concentrations of total lipids and cholesterol than the control.

According to the findings of this investigation, the administration of short peptide GBP can induce a substantial enhancement (*P* < 0.05) in the immunoglobulin concentrations in the blood of young broilers. Similarly, research conducted by Hou et al. [30] has demonstrated that antibodies in the polypeptides of soybean boost the animals' health and immunological response. Small peptides can stimulate phagocytic cells, lymphocytes, and immature splenocytes that might be responsible for the rise in blood immunoglobulins [60]. In addition, the *in ovo* administration with resveratrol resulted in increased levels of plasma IgG and IgM in newly hatched Mandara chicks [8]. Also, the levels of immunoglobulins (G, A, and M) in the blood of laying hens were significantly higher after the administration with short peptides [63]. Furthermore, it has been reported that bioactive peptides, like CBP, can operate on immunocytes, improving the immune system and disease resistance in animals [45]. In addition, Abd El-Azeem et al. [1] stated that delaying food availability negatively impacts the immune system, which supports the beneficial effects of *in ovo* feeding on immunity.

As a result of considerable levels of PUFAs in the lipids of chick embryo tissues, the development of antioxidant defenses is required. The high activity of the antioxidant defense system during embryogenesis could serve as a crucial adaptation mechanism for protecting the embryo tissues from the oxidation that occurs during the hatching process [21, 40, 53]. The results of the present work revealed an improvement in antioxidant status, which was proven by the increase in GSH level and SOD activity, and the reduction in MDA level of newly hatched chicks and 28-day-old chicks that were *in ovo* treated with CBP. The high levels of phenolic compounds found in CBPE (gallic acid, resveratrol, chlorogenic acid, catechin, hesperetin, naringenin, and syringic acid) significantly contribute to the potent antioxidant defense system. Our outcomes coincide with Amina et al. [8], who noted that *in ovo* administration with resveratrol significantly increased blood plasma levels of TAC and SOD and significantly decreased the MDA of the hatched chicks. As an additional point of interest, Abu Hafsa and Ibrahim [3] discovered that adding forty grams of grape seed to broiler diets led to a notable increase in antioxidant capacity and a decrease in the lipid peroxidation marker. The short peptide CBP was found to contain high concentrations of phenolic and flavonoid components, renowned for their antioxidant capabilities. The conclusion that can be drawn from this is that CBP can reduce reactive free radicals, which mitigates oxidative damage to tissues. This, in turn, can positively impact the immune system and growth performance of chicks that have been hatched.

## Conclusion

In general, the *in ovo* administration of the bioactive short peptide CBP through the amnion membrane at 18.5 days might be a valuable technique for boosting the growth performance of hatched broiler chicks, as well as for improving the health status in terms of blood lipid profile parameters, liver and kidney functions, immune response and antioxidant status. These beneficial effects on recently hatched chicks persisted until the market age and were most noticeable at a level of 2 mg CBP/egg.

## Data Availability

All the data generated or analyzed during this study are included in this published article.
